# U0126: Not only a MAPK kinase inhibitor

**DOI:** 10.3389/fphar.2022.927083

**Published:** 2022-08-25

**Authors:** Yijie You, Yunlian Niu, Jian Zhang, Sheng Huang, Peiyuan Ding, Fengbing Sun, Xuhui Wang

**Affiliations:** ^1^ Department of Neurosurgery, Xinhua Hospital Chongming Branch, Shanghai, China; ^2^ Department of Neurology, Xinhua Hospital Chongming Branch, Shanghai, China; ^3^ Department of Neurosurgery, Xinhua Hospital Affiliated to Shanghai JiaoTong University School of Medicine, The Cranial Nerve Disease Center of Shanghai JiaoTong University, Shanghai, China

**Keywords:** U0126, MAPK inhibitors, MAPK signal pathway, cancer, cancer therapy

## Abstract

U0126, as an inhibitor of the MAPK signaling pathway, is closely related to various biological processes, such as differentiation, cell growth, autophagy, apoptosis, and stress responses. It makes U0126 play an essential role in balancing cellular homeostasis. Although U0126 has been suggested to inhibit various cancers, its complete mechanisms have not been clarified in cancers. This review summarized the most recent and relevant research on the many applications of U0126 and described its role and mechanisms in different cancer cell types. Moreover, some acknowledged functions of U0126 researched in the laboratory were listed in our review. We discussed the probability of using U0126 to restain cancers or suppress the MAPK pathway as a novel way of cancer treatment.

## Background

**FIGURE 1:**
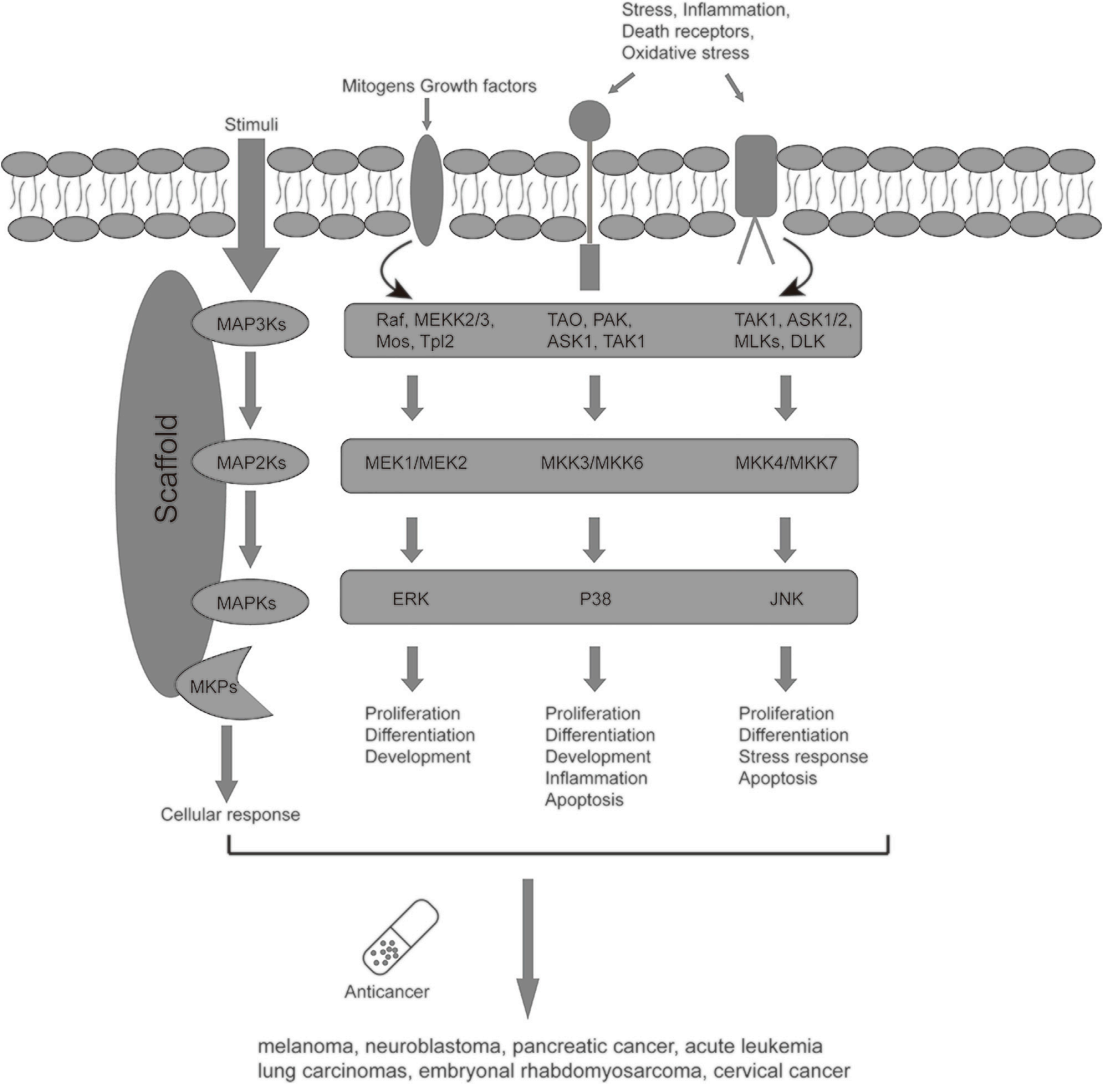
The known MAPK signaling pathways downstream target cell receptor signaling, working cooperatively to regulate cell physiology.

MAPK signaling pathway plays a vital role in cancer cell dissemination, proliferation, and drug resistance (De Luca et al., 2012). MAPK pathways were composed mainly of four families: 1) MAPK/ERK family or classical pathway; 2) Big MAP kinase-1(BMK-1); 3) c-Jun N-terminal kinase (JNK); 4) p38 signaling families (De Luca et al., 2012; Cossa et al., 2013) (Figure 1). In MAPK/ERK family, the carcinogenesis of ERK1/2 relates to upstream the activation of ERK1/2, which includes overexpression of RTKs (receptor tyrosine kinases) (Lu and Xu, 2006; Low and Zhang, 2016). Aberrant ERK1/2 activation is existed in various malignancies, including renal cell carcinoma, hepatocellular carcinoma, and gastric adenocarcinoma. The carcinogenesis of JNK mainly depends on the process of the phosphorylated c-Jun and activated AP-1 induced by JNK (Cellurale et al., 2011). JNK has two different proteins, JNK1 and JNK2, which make the JNK pathway dual role in cancer cells. Many studies have indicated that the JNK pathway can exert pro- and anti-oncogenic effects in different cancers and stages of cancer development (Wagner and Nebreda, 2009). In addition, JNK and p38 collectively have upstream activators and synergistically influence cancer cell survival (Svensson et al., 2011; Ruan et al., 2015). Recent studies have verified that increased phosphorylated p38 has been linked to various malignant tumors such as lung cancer, thyroid cancer, breast carcinoma, follicular lymphoma, and glioma. Nevertheless, the effect of p38 on cancer is complex and controversial at this stage (Wagner and Nebreda, 2009; Low and Zhang, 2016).

Over the last few years, U0126, as a signal transduction inhibitor of the MAPK (mitogen-activated protein kinase) pathway, has become the focus of relevant studies because of its impact on the development of malignancies (Ma et al., 2013). Many reports have shown that U0126 can inhibit tumor proliferation and enhance the anticancer effects of drugs or gene silencing treatments (Fukazawa et al., 2002; Wiesenauer et al., 2004; Marampon et al., 2006; Takayama et al., 2008; Marampon et al., 2009; Ito et al., 2010). U0126 mainly targets the RAF/MEK/ERK pathway in animal cells (Favata et al., 1998). It inhibits the activation of ERK1/2 by blocking the activation of the upstream MEK1/MEK2 and affecting p38 MAPK activity (Duncia et al., 1998; Wang and Studzinski, 2001; Hotokezaka et al., 2002). The RAF/MEK/ERK pathway and p38 pathway belong to MAPK signal pathways involving cell survival, differentiation, proliferation, apoptosis, and stress adaptation (Pagès et al., 1993; Matsumoto et al., 2002; Lefloch et al., 2008; Lefloch et al., 2009; Wu et al., 2011).

In this review, we described the role of U0126 in the MAPK signaling pathway and its biological activities, such as apoptosis, cell survival, and autophagy. At the same time, we concluded some new applications of this inhibitor from its molecular mechanism. Furthermore, we describe some well-recognized functions of U0126 stayed in the laboratory. Finally, we discussed the role of U0126 in different types of cancers and emphasized it as a potential ant-cancer drug that can improve the therapeutic effect on tumors.

## Structure and function of U0126

**FIGURE 2:**
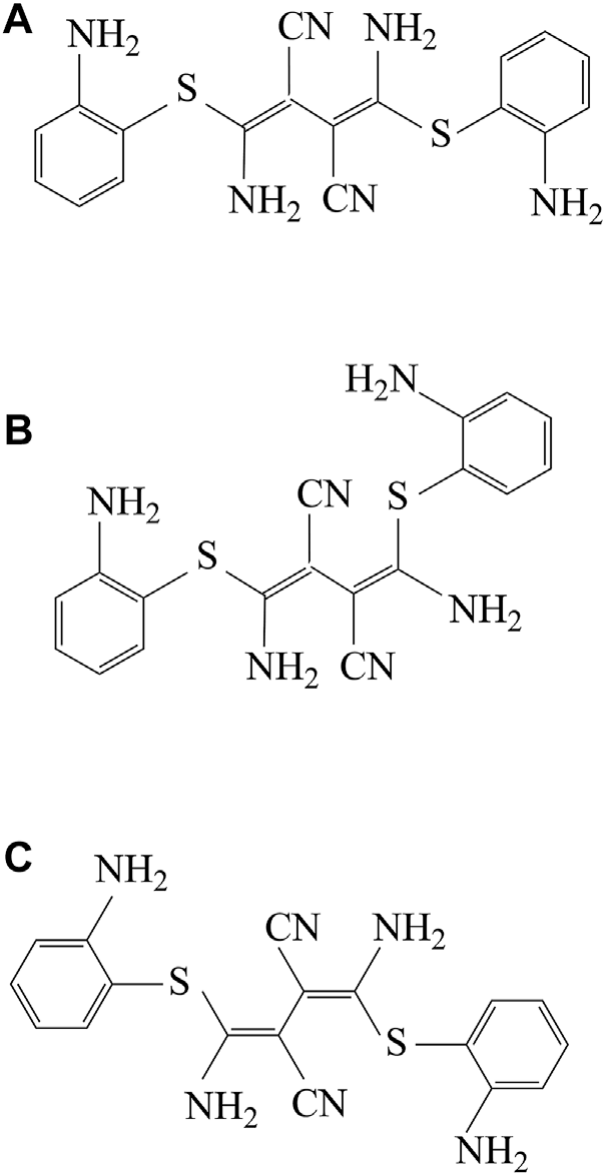
The structure and chemical characteristics of three isomers of U0126. **(A)** U0126, Z,Z-isomer; **(B)** U0126, Z,E-isomer; **(C)** U0126, E,E-isomer.

W. J. Middleton first synthesized U0126 in the late 1950s. U0126 can keep the crystalline state stable even for decades. There mainly include three possible isomers of U0126: Z, Z-isomer (Figure 2A); Z, E-isomer (Figure 2B); E, E-isomer (Figure 2C). As research progresses, Stephen et al. discovered that U0126 exerts its effects on cells *via* suppressing the activation of MEK1 (MAPK kinase 1; also known as MKK1) and not by blocking the activity. Therefore, U0126 has been widely researched in anti-tumor research as a MAPK inhibitor, but, in addition, U0126 also has biological effects in other aspects.

Our review of U0126 studies found that U0126 is more than just a MAPK inhibitor. We started from the anti-tumor aspect of U0126. We found that U0126 can affect multiple molecular signaling pathways, including MEK/ERK, JNK, KRAS, P44/42, JAK/STAT, PI3K/AKT/mTOR, and Ras/Raf/ERK signaling pathways. Moreover, U0126 can act on the following targets: ERK 1/2, MEK 1/2, C-JNK, μPA, MMP-9, P44/42, P21, p53, P27, and so on. The relevant results are summarized in Table 1. In the meantime, the roles of U0126 in several biological processes are described in detail below.

**TABLE 1:** U0126 as potential anticancer agents.

Tumor type	Tumor cell line	concentration	Duration	Mechanism	Molecular target	Biological activities	References
Melanoma	Human A375	5 μM/10 μM	24 h	MEK/ERK signaling pathway, JNK signaling pathway	↓p-ERK1/2, ↓p-MEK1/2, ↓c-Jnk,↓uPA, ↓MMP-9	↓ invasion, ↓ proliferation	Ge et al. (2002)
Neuroblastoma	SK-N-AS (S-type)	10 μM	24 h	MEK/ERK signaling pathway	↓p-ERK1/2	/	Eppstein et al. (2006)
	BE (2)-C (I-type)					↓ proliferation	
	SH-SY5Y (N-type)					/	
Pancreatic cancer	Mia PaCa-2	2.5–80 μM (Mix = 20 μM)	15 min	MAPK signaling pathway	↓p-ERK, ↓p-MEK1/2	↓ proliferation	Guo et al. (2015)
				KRAS signaling pathway			
	BxPC-3	0–30 μM (IC50 = 30 μM)	72 h	MEK/ERK signaling pathway	↓p-ERK	↓ proliferation	Yip-Schneider and Schmidt, (2003)
	PANC-2	0–30 μM (IC50 = 25 μM)				↓ proliferation	
	Mia PaCa-2	0–30 μM (IC50 = 10 μM)				↓ proliferation	
Gallbladder cancer	NOZ cells	0,1, 5, 10, 50, 100 μM	48 h	/	/	↓ proliferation	Horiuchi et al. (2003)
Acute leukemia	KG1a	50 μM	48 h	p44/42 (MAPK) signaling pathway	↓p44/42	↓ proliferation, ↑apoptosis	James et al. (2003)
	HEL						
	M-07e						
	TF1						
	THP-1						
Lung carcinomas	RAW264.7	5 mg/100 g	10–40 days	JAK/STAT3 signaling pathway, PI3K/AKT signaling pathway	↑IFN-γ	↓ proliferation	Ma et al. (2015)
	EL-4						
	A549	1, 3, 5, 10, 20 μM	72 h	PI3K/AKT/mTOR signaling pathway, Ras/raf/ERK signaling pathway	p21, p53, p27, cyclin D1, cyclin E1	↓ proliferation, ↑apoptosis, ↓cell cycle (G0/G1)	Zou et al. (2012)
	H460						
Embryonal rhabdomyosarcoma	RD	25 or 50 μmol/kg	5 weeks	MEK/ERK signaling pathway	↓C-Myc	↓ proliferation	Marampon et al. (2009)
	TE671						
Cervical cancer	Hela	1, 2, 5, 10, 20, 30 μM	4 h	ERK signaling pathway, JAK-STAT signaling pathway	↓p-ERK1/2	↓ invasion, ↓ proliferation, ↑apoptosis,↓cell cycle (G0/G1)	Ye et al. (2017)

Mix, Maximum inhibitory concentration; IC50, 50% inhibitory concentration; ↑, Promotion; ↓, Inhibition.

### Cell growth and differentiation

Many findings have supported that chemical inhibitors suppressing signal transduction are potent tools in exploring signaling pathways. As a MAPK inhibitor, U0126 is widely used in investigating what pathways are involved in cell responses, such as growth and differentiation. It has been demonstrated that U0126 can inhibit MEK leading to an apparent decrease of phosphorylated ERK, accelerating the differentiation of RAW264.7 cells into osteoclast-like cells (Hotokezaka et al., 2002). Moreover, Xu et al. (2015) demonstrated that U0126 could promote osteogenic differentiation of rat MSCs model by activating the BMP/Smad pathway.

### Apoptosis

Apoptosis is an important mode of cell death that is no longer needed or is a pathologic status to the organism, including nuclear chromatin condensation, cell shrinkage, and caspase activation (Patel et al., 1996; Thornberry, 1998). U0126 is a potent anti-apoptotic agent. Jo et al. found that U0126 decreases apoptosis and the activation of caspase 3 through inhibition of ERK1/2, suggesting pretreatment of U0126 has significant functional and histologic protection to attenuate cisplatin-induced renal injury (Jo et al., 2005). Interestingly, U0126 can also induce apoptosis to inhibit the proliferation of tumors. For example, U0126 can cause apoptosis in leukemic blast cells, especially in the KG1a cell line (Kerr et al., 2003). Accordingly, U0126 can potentiate or antagonize apoptosis, depending on the drug or the target cells. It is still indistinct whether these different regulations on apoptosis are due to a direct effect of U0126 or whether it is the only result of the inhibition of the MAPK pathway, or whether there exist other pathways. Therefore, further studies are needed to explore the existence of potential mechanisms of U0126 in the apoptosis pathway.

### Autophagy

Autophagy is a process of self-degradation. Autophagosome degradation removes damaged cell organelles and misfolded or aggregated proteins (Glick et al., 2010). This pathway is important for limiting tumor initiation because it can inhibit genomic instability and oxidative stress (Eskelinen, 2011). Cells can use this process to balance energy sources in response to nutrient stress such as serum starvation or glucose (Glick et al., 2010). Interestingly, many studies have reported that U0126 is involved in suppressing autophagy. Wang et al. verified that U0126 inhibits cisplatin-induced autophagy in HEI-OC1 and cochlear hair cells (Wang et al., 2021). Moreover, Wang et al. found that U0126 reduces ischemia/reperfusion-induced autophagy in the myocardium (Wang et al., 2016). However, there is no evidence of the activation of autophagy by U0126. Due to limited data, the effect of U0126 on autophagy needs further research, especially in different cells.

### Inflammation

Inflammation occurs when tissues respond to injury. The various cell types’ expression and diverse mediators’ reactions both play a role in inflammation. Significantly, The ERK 1/2 pathway, as the most extensively occurred member of the MAPK pathway, is related to inflammation (Mohammad et al., 2013). U0126, a potent and selective MAPK inhibitor, can decrease ERK 1/2 activation. According to these theories, many studies have further verified that U0126 has anti-inflammatory effects. For example, U0126 reduces diabetes-induced upregulation of MMP-9 and biomarkers of inflammation in the retina (Mohammad et al., 2013). In the asthma mouse model, U0126 attenuates pulmonary eosinophilia, OVA-induced Th2 cytokine production, serum IgG1, IgE synthesis, mucus hypersecretion, and AHR to restrain allergic airway inflammation (Duan et al., 2004).

## U0126 and cancer

U0126 has been found to play an antiproliferative role in cancers. Interestingly, there are different antitumor mechanisms of U0126 in each tumor. To achieve clear comprehension, we summarized existing studies and listed known antitumor mechanisms of U0126 in the following section (Table 1).

### U0126 and melanoma

Tumor cell invasiveness is a multistep process including cell adhesion, matrix proteolysis, and cell migration. The extracellular matrix’s degradation needs invasive proteases secreted by the invading cells, including uPA (urokinase plasminogen activator) and MMP (matrix metalloproteinase) (Aguirre Ghiso et al., 1999; Jesionowska et al., 2015). In human melanoma, U0126 not only inhibits phosphorylation of MEK 1/2 and ERK 1/2 but also decreases the expression of c-Jun, a significant component of the transcription factor AP-1 (Ge et al., 2002). Because the gene promoter regions of uPA and MMP-9 contain AP-1, the decrease of c-Jun suppresses the expression of uPA and MMP-9. Therefore, U0126 can significantly inhibit melanoma invasion *via* decreasing uPA and MMP-9 concentrations (De Petro et al., 1998; Lakka et al., 2000).

### U0126 and neuroblastoma

There are three unique cell phenotypes in neuroblastoma cells: neuroblastic (N-type), intermediate (I-type), and substrate-adherent (S-type) (Ross et al., 2003). Those cell types differentiate into another type in culture, and the proportions of each type of tumor are different (Ross and Spengler, 2004). Because cell growth and differentiation involve the MAPK pathway, many researchers believe in the feasibility of MAPK-targeted therapies in tumors (Giroux et al., 1999; Mattingly et al., 2001; Ross and Spengler, 2004). Eppstein et al. (2006) found that all three cell types exhibit the expression of p-ERK decreased after processing by MEK inhibition. However, only I-type cells exhibit significantly decreased proliferation with U0126 treatment. Although U0126 has the promise of targeting I-type cells, neuroblastoma treatments may need to combine agents against N-type and S-type cells. It may be a new point to explore tumor-targeted therapeutic strategies further.

### U0126 and pancreatic cancer

Pancreatic cancer is a common malignancy worldwide, with a median survival time of fewer than 6 months (Hidalgo, 2010). Several targeted therapies have been used in researching pancreatic cancer (Vaccaro et al., 2011). Pancreatic cancer cell growth depends on the activity of the mutated KRAS gene. Therefore, silencing the KRAS gene can control pancreatic cancer cell line proliferation (Réjiba et al., 2007). It makes the components in the KRAS pathway become promising targets for identifying novel therapies (McCubrey et al., 2007). In addition, the ERK signaling pathway is not regulated in pancreatic carcinoma cells despite KRAS gene expression, and the reason is that increased MKP-2 (MAP kinase phosphatase-2) inactivates ERK. The results showed that both KARs gene expression and the MAPK-ERK pathways are involved in the occurrence of pancreatic cancer. Similarly, if the MEK-ERK signaling pathway is necessary for the growth of the pancreatic cell, it may be a potential therapeutic target alone or with other cellular pathways (Yip-Schneider et al., 1999; Yip-Schneider et al., 2001).

Interestingly, these hypotheses have been confirmed in subsequent studies. For example, U0126 effectively controls pancreatic cancer cell line proliferation *via* targeting the downstream effectors of KRAS signaling in a zebrafish xenotransplantation model (Guo et al., 2015). In addition, Yip-Schneider and Schmidt (2003) confirmed that U0126 dependently inhibits the growth of three human pancreatic carcinoma cell lines (PANC-1, BxPC-3, and MIA PaCa-2).

### U0126 and gallbladder cancer

It is challenging to diagnose gallbladder cancer in clinical practice because the symptoms and the manifestations of gallbladder cancer are similar to benign gallbladder disease. Therefore, most cases of gallbladder cancer are found at an advanced stage, accompanied by metastases to the lymph nodes, invasion of the liver, and distant organs. Most of the time, the tumor is unresectable despite radical surgery (Mizuguchi et al., 1997; Levin, 1999). Oncogenic mutation of KRAS is associated with gallbladder carcinogenesis (Watanabe et al., 1994; Ajiki et al., 1996; Hidaka et al., 2000). Activation of KRAS can induce the constitutive activation of the MAPK pathway and PI3K-AKT pathway, which rapidly develops the growth of the gallbladder epithelium (Leevers and Marshall, 1992; Thomas et al., 1992; Rubio et al., 1997). Furthermore, point mutation of P53 is also related to gallbladder cancer carcinogenesis (Hanada et al., 1997; Watanabe et al., 1999). Therefore, U0126 as a MAPK inhibitor has the potential to inhibit gallbladder cancer proliferation. Moreover, a recent study has verified that U0126 observably prolongs the survival duration of mice with gallbladder tumors. The major organs such as kidneys, liver, small intestine, colon, stomach, brain, lungs, and heart do not have apparent histopathological abnormality after U0126 treatment in mice bearing gallbladder cancer cells with KRAS mutation (Horiuchi et al., 2003). However, the underlying mechanisms of U0126 inhibiting gallbladder cancer need to be further explored.

### U0126 and acute leukemia

The control of cell proliferation, differentiation, and apoptosis depends on the balance between a series of signaling cascades. In acute leukemia, this delicate balance is frequently deranged. Blockade of proliferative pathways by inhibiting MEK is growth inhibitory or pro-apoptotic in some acute myeloblastic leukemia (AML) cell lines and some AML patients (Berra et al., 1998; Jarvis et al., 1998; Milella et al., 2001; Morgan et al., 2001; Baines et al., 2002). It has been reported that the MEK inhibitor U0126 induces significant levels of apoptosis in three acute leukemia cell lines, KG1a, THP-1, and M-07e (James et al., 2003). Although the sensitivity of different cell lines is variable, U0126 seems to offer a potential alternative to standard chemotherapeutic agents in treating acute leukemia.

### U0126 and lung carcinomas

A recent study has reported that U0126 inhibits chemically-induced pulmonary carcinomas’ growth and improves tumor-free survival rates in mice with inoculated lung carcinomas. Among them, the antitumor effect of U0126 mainly depends on the activation of IFN-γ production (Ma et al., 2015). IFNs (Interferons) are a family of pleiotropic cytokines including three major groups: Type I, Type II, and Type III IFNs (Maher et al., 2007). IFN-γ is the sole member of Type II IFN, and it has multiple biological functions in defense and immune systems, just like the antiviral, antimicrobial, antiproliferative, and antitumor activity (Schroder et al., 2004; Schoenborn and Wilson, 2007). However, more and more evidence has shown that IFN-γ can also induce tumor progression. It makes the role of IFN-γ in regulating antitumor immunity appear complex and paradoxical. Related literature reports that IFN-γ can promote lung cancer progression *via* the JAK/STAT3 signaling pathway and PI3K/AKT signaling pathway in lung carcinomas (Zhang et al., 2017). Thus it can be seen that the relationship between U0126 and INF-γ and the effect of INF-γ on lung cancer are skeptical. Therefore, we think that the role of U0126 in lung cancer is unclear.

It is worth noting that if U0126 has an inhibitory effect on lung cancer, it may be associated with inhibition of cell cycle and proliferation. The explanation is as follows: U0126 can exert its effects on G0-G1 arrest *via* up-regulating p21, p53, and p27. Meanwhile, cyclin D1 and cyclin E1 are down-regulation (Zou et al., 2012).

### U0126 and embryonal rhabdomyosarcoma

In childhood, RMS (malignant tumors of skeletal muscle rhabdomyosarcoma) is the most common soft-tissue sarcoma (Merlino and Helman, 1999). C-Myc, N-Myc, and MYCL1 play an important role in human cancer (Adhikary and Eilers, 2005). In conditional transgenic models on Myc inactivation, tumors can regress (Jain et al., 2002; Shachaf et al., 2004). Ras mutation activating MEKs and ERKs occurs in various tumors (Kohno and Pouyssegur, 2003; Faivre et al., 2006; Liu et al., 2007). C-Myc is targeted by ERKs that stabilize C-Myc, whereas GSK-3β induces C-Myc degradation (Sears et al., 2000; Yeh et al., 2004). Ras activation can induce chronic MEK/ERK activation and phosphatidylinositol 3-kinase/AKT-mediated GSK-3β inactivation leading to C-Myc aberrant accumulation (Bachireddy et al., 2005). According to the above basic theory, Marampon et al. used the MEK/ERK inhibitor U0126 and embryonal rhabdomyosarcoma cell line-xenotransplanted mice to verify whether MEK//ERK inhibitions affect C-Myc protein level and growth of RMS tumor. They found that U0126 significantly reduces RMS tumor growth *via* disrupting C-Myc (Marampon et al., 2009). Thus, U0126 could be used in a signal transduction-based therapy for RMS and warrants testing in RMS trials.

### U0126 and cervical cancer

Cervical cancer is a common malignant tumor in females (Chan and So, 2015). ERK1/2 signaling pathway plays an essential role in cervical cancer differentiation (Chang et al., 2014). ERK 1/2 is expressed in cervical cancer tissues in cytoplasm and nuclei (Kake et al., 2017). Moreover, ERK can facilitate cancer cell growth *via* promoting cell movement from the G1 phase to the S phase. U0126, an ERK inhibitor, can decrease cell content in the S phase, which may restrain breast cancer proliferation by blocking the cell cycle (Zhao et al., 2009). In addition, U0126 can induce apoptosis to suppress cervical cancer. The induced-apoptosis mechanism of U0126 in cervical cancer relates to the inhibition of the ERK signaling pathway and the suppression of the JAK-STAT pathway (Zhao et al., 2009; Ye et al., 2017) .(de Tomaso Portaz et al., 2015)

## Limitations and prospects

Currently, the effects of U0126 are limited in treating human cancers unless particular cancer proliferates mainly relies on the MAPK signaling pathway. Moreover, the anticancer effects of U0126 often depend on cytostatic rather than cytotoxic. Although it is an effective anticancer agent in a single treatment setting, existing research is restricted to a laboratory experiment state.

However, many studies have confirmed that U0126 may be more effective when combined with chemotherapies or radiotherapies (Yacoub et al., 2003; Gao et al., 2005; Ito et al., 2010). It means U0126 could overcome the resistance to chemotherapeutic drugs (Shi et al., 2014). Thus, combination therapy with either a traditional drug/physical treatment or U0126 is also a meaningful way to improve the effectiveness and usefulness of U0126.

As the technology develops, more and more data support the role of molecular signaling pathways in cancer biology. While on a single tumor, molecular “tailored” treatment is still the most ambitious goal, but to build a new, based on the combination mechanism, may bypass the escape mechanism, and in patients with relatively not choose groups to overcome the resistance of single channel inhibitors, seems to have in our ability range, and possible treatment of substantial progress soon. In general, MEK inhibitors are well tolerated, with only rash edema and transient blurred vision being common side effects. Importantly, plasma concentrations of each compound are sufficient to inhibit MEK *in vitro* tumor tissues. Although the concentration of U0126 is relatively high *in vitro*, it is still an optimistic assumption that subsequent studies can combine U0126 with other drugs to achieve the maximum tumor suppressive effect at the minimum concentration and apply it in clinical practice.

Researchers and doctors sometimes have a purposely narrow view of a particular topic. For example, cancer researchers think that U0126 can suppress the growth of cancer cells. Nevertheless, U0126 may also help treat inflammatory and tissue injury with abnormal cellular proliferation (Clemons et al., 2002; Cho et al., 2004; Christensen et al., 2019). These research topics, such as ischemic brain injury (Farrokhnia et al., 2008), myocardial ischemia (Wang et al., 2016), and asthma (Duan et al., 2004), significantly improve the potential clinical uses of U0126.

In summary, U0126, a drug discovered a long time ago, has been reported to have anti-tumor effects, but it may not be paid too much attention due to force majeure reasons. With the development of science and technology, we do not want U0126 to be buried in history. Therefore, in this article, we will review its functions and mechanisms again, hoping to arouse people’s attention. If combined with the existing new technology, U0126 can solve the previous legacy problems and become a powerful tool not only for anti-tumor but also for treating other diseases. We believe it will provide hope for the majority of patients.

## Conclusion

In this review, we described that U0126 is an inhibitor in the cell proliferation of many cancers, mainly through its role in blocking the MAPK pathway. If one tumor depends on the MAPK pathway, it may be sensitive to U0126. In addition, U0126 will only exhibit antitumor effects combined with cytotoxic chemotherapeutic drugs or radiation. However, recent studies also describe other mechanisms of U0126, which makes U0126 more critical to research how it can become a drug of anticancer therapies. At the same time, U0126 may also be considered for development in treating other diseases due to its ability to affect apoptosis, autophagy, and inflammation.
